# Uterine Artery Embolisation: A Saviour for Central Placenta Previa

**DOI:** 10.7759/cureus.47168

**Published:** 2023-10-17

**Authors:** Preeti Gattani, Priya Nair, Sandeep Khandare, Rasika D Zade

**Affiliations:** 1 Obstetrics and Gynaecology, Datta Meghe Medical College, Datta Meghe Institute of Higher Education & Research (DU), Nagpur, IND; 2 Obstetrics and Gynaecology, Datta Meghe Medical College, Datta Meghe Institute of Higher Education and Research (DU), Nagpur, IND

**Keywords:** maternal mortality, obstetrics hysterectomy, prophylactic interventional procedure, uterine artery embolization, placenta accreta, placenta previa

## Abstract

Central placenta previa is generally managed by caesarean section, uterotonic drugs, hemostatic sutures, and hemostatic balloon tamponade, and it usually ends in peripartum hysterectomy, with an incidence of 4% along with an increased incidence of maternal and neonatal morbidity or mortality. Selective uterine artery embolization (UAE) helps to prevent these complications while preserving future fertility. A 32-year-old patient was diagnosed as a case of a previous section with central placenta previa with accreta at 38 weeks and was planned for an elective caesarean section. A prophylactic selective bilateral internal iliac artery cannulation under sonographic guidance and subsequent cannulation and embolisation of bilateral uterine arteries under fluoroscopic guidance was done. It reduced the massive blood transfusions and operative time, preserved fertility without any maternal or foetal morbidity or mortality, and no other complications were reported.

## Introduction

The central placenta previa is defined as the placenta completely covering the cervical os and is one of the risk factors for increasing maternal morbidity and mortality. The estimated incidence of placenta previa is one in 200 pregnancies [1] or three to five in 1,000 pregnancies [2]. But in modern obstetrics, the incidence is rising because of increased risk factors like multiple pregnancies, previous caesarean sections, advanced maternal age, and assisted reproductive techniques [3]. Placenta previa is associated with life-threatening antepartum, intrapartum, and postpartum haemorrhage, which are traditionally managed by caesarean section, uterotonic drugs, hemostatic sutures, and hemostatic balloon tamponade and usually end in obstetric hysterectomy, maternal and neonatal morbidity, or mortality [1]. Postpartum haemorrhage due to placenta previa accounts for 30% of maternal deaths in Asia. Newer approaches have been used to reduce blood loss, like combining endovascular techniques with surgery. In 1979, uterine artery embolisation was used for the first time to treat postpartum haemorrhage, with a 90% success rate and good clinical outcome [4]. Selective uterine artery embolisation (UAE) helps control the bleeding and prevents postpartum haemorrhage and, eventually, peripartum hysterectomy and maternal mortality [5].

Here, we discuss a case of central placenta previa grade IV with suspected accreta with prophylactic sonographic-guided internal iliac artery cannulation to reduce foetal radiation exposure and subsequent bilateral selective UAE under fluoroscopic guidance, thus preventing massive blood transfusions, preserving the uterus without maternal and foetal morbidity, and reducing mortality without any complications.

## Case presentation

**Figure 1:**
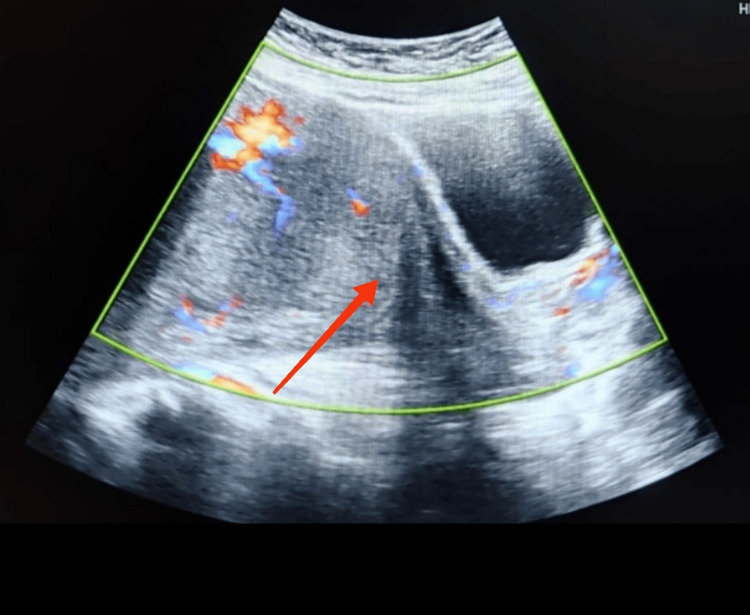
Ultrasonography of central placenta previa The arrow shows the placenta completely covering the cervical os.

A 32-year-old, third gravida with previous term delivery by caesarean section for transverse lie was diagnosed as a case of central placenta previa with suspicion of placenta accreta at 33 weeks of gestation on ultrasonography. Figure 1 shows the ultrasonographic finding of the central placenta previa.

The patient was admitted to the antenatal ward for further evaluation. The abdomen examination showed uterine fundal height at 32 weeks with a transverse lie, the uterus was relaxed, and the fetal heart rate was regular. Routine blood investigations were within normal limits. An MRI was done for suspected placenta accreta, which showed grade four placenta previa, anteriorly reaching above the fibrous scar. The patient was given two doses of injection betamethasone 24 hours apart for the lung maturity of the foetus. The patient was managed conservatively until 38 weeks of gestation, with strict monitoring of vitals and a daily foetal movement count. She was given a rescue dose of 12 mg of betamethasone. Two units of blood were reserved. An elective caesarean section was planned at 38 weeks of gestation. The patient was discussed with an interventional radiologist, and it was decided to do a prophylactic internal iliac artery balloon cannulation under sonographic guidance to reduce the risk of radiation to the foetus. Later, selective bilateral uterine artery cannulation and, subsequently, embolisation under fluoroscopic guidance were considered. The patient was explained regarding the whole procedure and the risks, and written informed consent was obtained.

**Figure 2:**
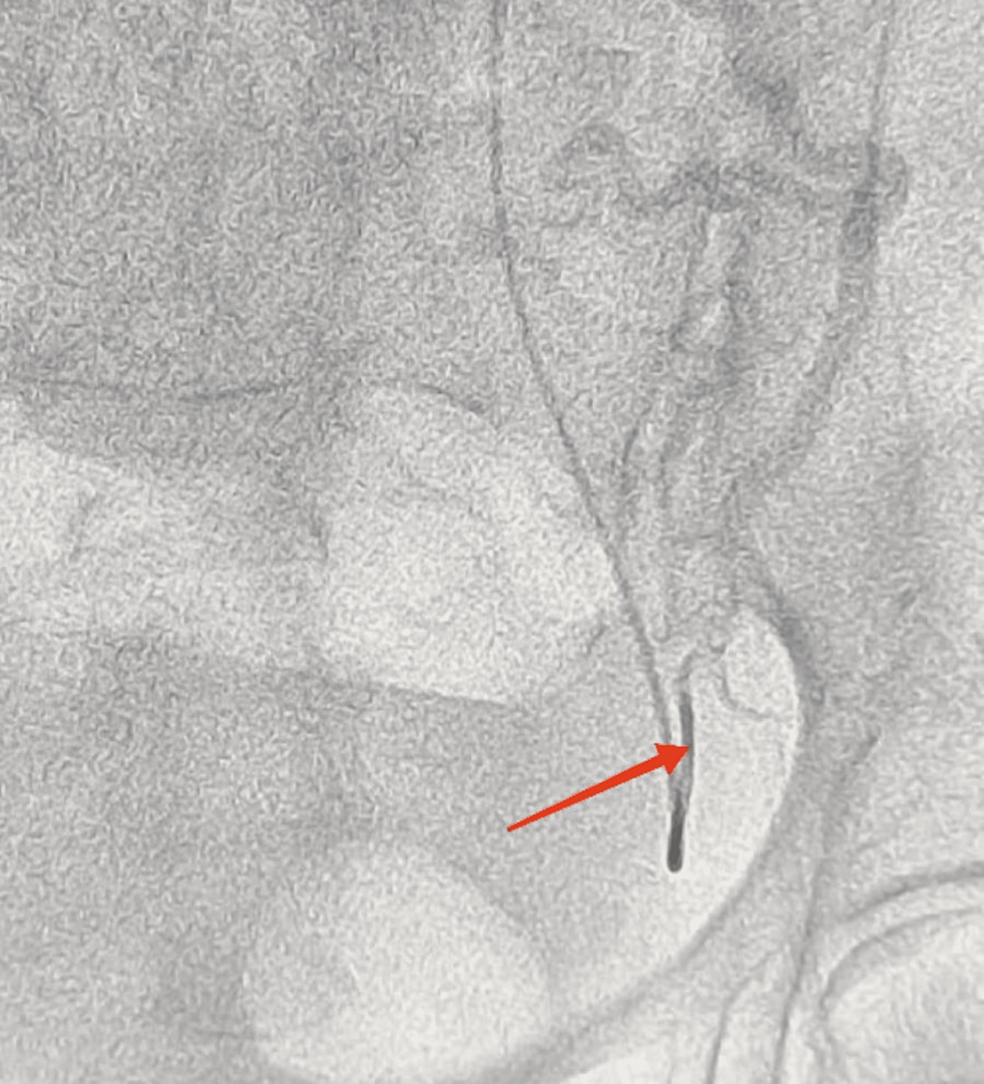
Left uterine artery cannulation The arrow shows the left uterine artery post cannulation.

On the day of the caesarean section, just before the operation, bilateral internal iliac artery cannulation was done by 5-French sheaths under sonographic guidance, and selective uterine artery cannulation under fluoroscopic guidance was done. Figure 2 shows the uterine artery cannulation before the caesarean section.

**Figure 3:**
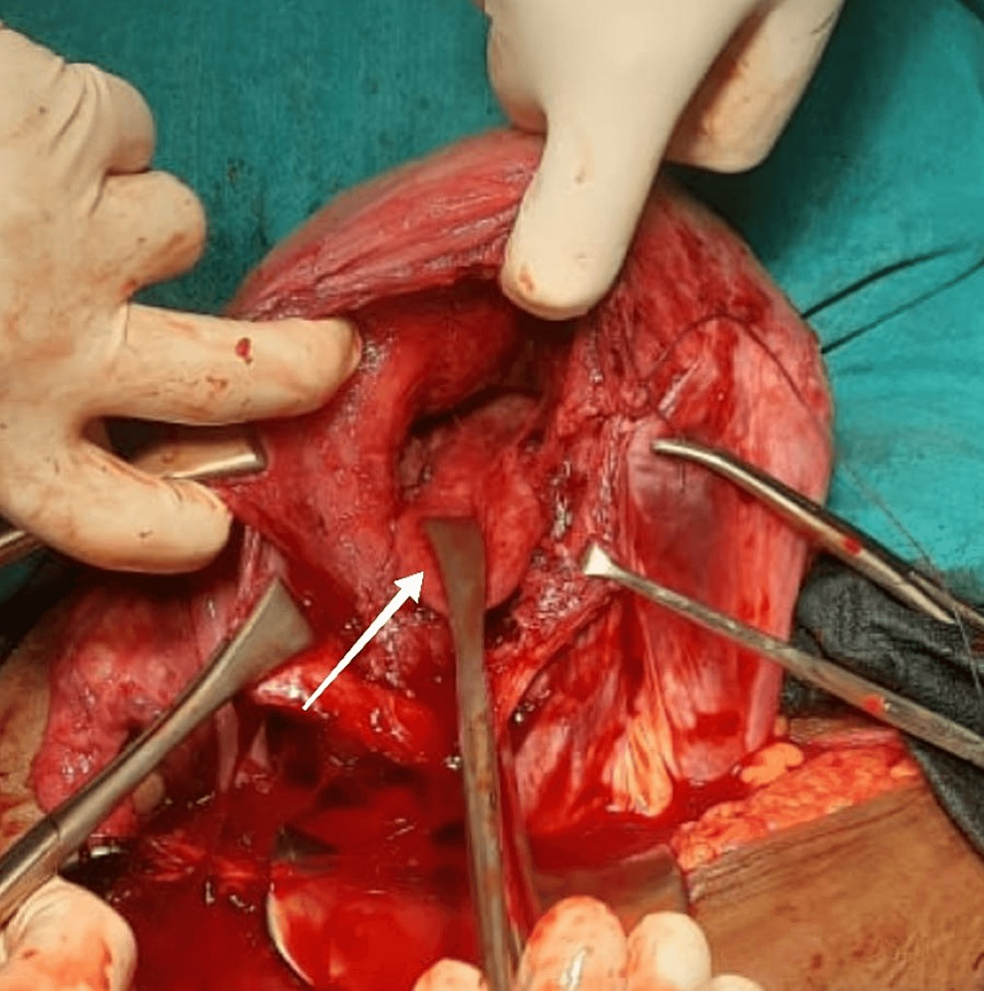
Intraoperative subseptate uterus The arrow shows the septum arising from the fundus reaching the incision site.

A caesarean section was performed, and the placenta was cut through to deliver the foetus. A full-term male foetus was extracted by breech with a birth weight of 2800 grams. The baby cried immediately after birth. After the baby was born, bilateral UAE was done under fluoroscopic guidance using 300-500 micrograms of polyvinyl alcohol (PVA) particles. The uterus was blanched entirely, and the placenta was removed with minimal bleeding. The uterus was subseptate. Figure 3 shows the intraoperative subseptate uterus.

Uterine closure and abdominal closure were uneventful, with minimal intraoperative blood loss. The total operative time was approximately 25 minutes. The patient was strictly monitored for the first 12 days in the hospital. Her postoperative period was comfortable without postpartum haemorrhage. The patient developed one fever spike on the seventh postoperative day, managed by a single dose of paracetamol. The rest of the postoperative period was uneventful, without any complications. Her stitches were removed on the tenth day, and the wound was healthy. The patient was again followed up after two weeks and six weeks without complications. The patient tolerated the procedure very well.

## Discussion

Placenta previa is rising due to multiple factors, which leads to life-threatening bleeding, posing a severe threat to the mother and baby [1,6]. Based on recent studies, the application of vascular interventional therapy in such cases can reduce bleeding and massive blood transfusions and preserve future fertility [6].

Delivery in cases of placenta previa precipitates severe postpartum haemorrhage due to unsuccessful forcible attempts at removal of the placenta. This leads to major complications, including massive blood transfusions, disseminated intravascular coagulopathy, acute renal failure, infectious morbidities, acute respiratory distress syndrome (ARDS), and infertility. Since the onset of UAE in gynaecological pathologies like fibroids and adenomyosis [1], very few cases of prophylactic UAE in managing obstetric cases of placenta previa to minimise blood loss have been reported [5-7].

The conventional management of a case of central placenta previa with accreta involves a caesarean section. It ends up either in an internal iliac artery ligation or an obstetric hysterectomy, thus increasing morbidity, future fertility, and mortality. Bilateral internal iliac artery ligation is associated with various complications, like claudication of the buttocks and thigh and damage to the ureter due to an interruption of blood supply to the ureters and buttocks. A planned caesarean hysterectomy at the time of the caesarean delivery has been the standard of care and has been a life-saving procedure. But this causes future infertility. A minimally invasive medical therapy of selective UAE by PVA particles selectively embolises the uterine artery, giving the desired effect. This also prevents the complications of bilateral internal iliac artery ligation [8].

In a similar case reported by Radaelii et al., prophylactic UAE was done by uterine artery cannulation and embolisation before the delivery of the baby. However, in our case, we performed a bilateral internal iliac artery cannulation through a femoral approach under sonographic guidance to minimise the effect of radiation exposure on the fetus. Selective bilateral uterine artery cannulation was done under fluoroscopic guidance. In the case reported by Radaelii et al., embolisation of the uterine artery was done before the caesarean section 1]. In this case, bilateral UAE with 300-500 micron PVA particles was done after the delivery of the baby to avoid foetal heart rate variations and foetal stress. There was minimal intraoperative and postoperative blood loss.

In a similar case reported by Qiang et al., prophylactic UAE was done, and they concluded that UAE effectively reduced the blood loss, the need for blood transfusion, and the incidence of disseminated intravascular coagulation (DIC) among women with placenta previa complicated by accreta. However, they did not find any significant differences in hysterectomy incidence or duration of ICU stay between the groups [6].

Hae Jeng Lim et al. reported two cases of central placenta previa in which UAE was done after the delivery of the baby to prevent foetal radiation. In both cases, the uterus was saved, but the blood loss required massive blood transfusions and fresh frozen plasma [9].

In a similar article by Abdallah et al., prophylactic bilateral embolisation in three cases of placenta previa with accreta was done. They concluded that the conservative approach reduced peripartum hysterectomy, injury to adjacent organs, and massive blood transfusions while conserving fertility. However, they followed a conservative management style where the placenta was left in situ, followed by methotrexate [10].

## Conclusions

In patients with anticipated severe blood loss, preventive placement of a balloon catheter and UAE can effectively reduce the blood loss, shorten the operative time, prevent multiple transfusions, prevent peripartum hysterectomy, and reduce hospital stay. Thus, a vascular interventional approach that involves selective UAE in patients with central placenta previa, mainly when associated with accreta, should be preferred to conserve fertility and reduce maternal and foetal morbidity and mortality.
